# Targeting choroid plexus epithelium as a novel therapeutic strategy for hydrocephalus

**DOI:** 10.1186/s12974-022-02500-3

**Published:** 2022-06-17

**Authors:** Yijian Yang, Jian He, Yuchang Wang, Chuansen Wang, Changwu Tan, Junbo Liao, Lei Tong, Gelei Xiao

**Affiliations:** 1 Department of Neurosurgery, Xiangya Hospital, Central South University, No. 87, Xiangya Road, Changsha, Hunan 410008 People’s Republic of China; 2 Diagnosis and Treatment Center for Hydrocephalus, Xiangya Hospital, Central South University, No. 87, Xiangya Road, Changsha, Hunan 410008 People’s Republic of China; 3 National Clinical Research Center for Geriatric Disorders, Xiangya Hospital, Central South University, No. 87, Xiangya Road, Changsha, Hunan 410008 People’s Republic of China; 4 Department of Pediatrics, Xiangya Hospital, Central South University, Changsha, Hunan 410008 People’s Republic of China

**Keywords:** Choroid plexus epithelium, Cilia, Hydrocephalus, Cerebrospinal fluid, Pathogenesis

## Abstract

The choroid plexus is a tissue located in the lateral ventricles of the brain and is composed mainly of choroid plexus epithelium cells. The main function is currently thought to be the secretion of cerebrospinal fluid and the regulation of its pH, and more functions are gradually being demonstrated. Assistance in the removal of metabolic waste and participation in the apoptotic pathway are also the functions of choroid plexus. Besides, it helps to repair the brain by regulating the secretion of neuropeptides and the delivery of drugs. It is involved in the immune response to assist in the clearance of infections in the central nervous system. It is now believed that the choroid plexus is in an inflammatory state after damage to the brain. This state, along with changes in the cilia, is thought to be an abnormal physiological state of the choroid plexus, which in turn leads to abnormal conditions in cerebrospinal fluid and triggers hydrocephalus. This review describes the pathophysiological mechanism of hydrocephalus following choroid plexus epithelium cell abnormalities based on the normal physiological functions of choroid plexus epithelium cells, and analyzes the attempts and future developments of using choroid plexus epithelium cells as a therapeutic target for hydrocephalus.

## Background

Hydrocephalus is a common neurological disease with complex etiology and frequently unsatisfactory outcome. It can develop at all ages, with infants and the elderly as the most frequent groups, with congenital hydrocephalus in infants and idiopathic normal pressure hydrocephalus (iNPH) in the elderly [1].

It is believed that under the action of multiple factors, cerebrospinal fluid (CSF) circulation becomes impaired and CSF accumulates excessively [2], causing enlargement of the ventricular system, damaging the cerebral vasculature [3] and injuring the neurological function of the patient. In Alzheimer's disease, altered expression of type I and II interferons which is involved in the recruitment of immune cells to the central nervous system [4], increases β-amyloid (Aβ) in the CP, leading to damage of capillary and interstitial fibrosis [5]. In addition, Aβ causes increased expression of pro-inflammatory cytokines and matrix metalloproteinases, which in turn leads to downregulation of TJ proteins, resulting in disruption of the blood–CSF barrier (BCSFB), making it impossible to clear Aβ [6]. In most cases, the infection is a sequel to inflammatory diseases such as meningitis, and the inflammatory response of the body to fight the infection also stimulates the secretion of CSF from the choroid plexus epithelium, causing the accumulation of CSF. In addition, it has been demonstrated that the choroid plexus continues to serve as a long-term reservoir for the virus in time to receive antiviral therapy and is the pathway for the virus to enter the brain from the blood [7]. There is no significant difference between choroid plexus (CP) papilloma and CP carcinoma., both of which exhibit choroidal hypertrophy and diffuse enlargement of the CP on the basis of a normal histological pattern [8]. This change, in turn, allows for a much higher secretion of CSF and thus the development of secondary hydrocephalus [9]. The pathological features are accumulation of CSF in the ventricular system and subarachnoid space [10]; imaging features are enlargement of the lateral cerebral sulcus and narrowing of the intraparietal sulcus.

In the past perception, it was believed that the lymphatic system did not exist in the brain. However, a study in 2000 found the existence of a system of intracranial material removal in the brain similar to the lymphatic system, and named it the glymphatic system [11]. Its essence is that CSF from the subarachnoid space is driven into the perivascular spaces of the arteries on the surface of the brain. It then flows into the brain parenchyma via aquaporin (AQP) on astrocytes, resulting in interstitial fluid (ISF), which in turn flows into the perivascular spaces of the veins. Finally, it can be eliminated by blood circulation. When the removal of substances from the glymphatic system is reduced, it leads to hydrocephalus [12].

Through the efforts of many researchers, the pathogenesis of hydrocephalus is being completed, but it has not been fully explained [13]. The widely accepted and applied theories are the circulation theory of CSF and the osmotic pressure theory of CSF. The basic contents of the circulation theory of CSF are that CSF secreted by the choroid plexus epithelium (CPE) flows along a specific pathway and is finally absorbed by the venous sinuses. This theory explains the imbalance between the production of CSF by the CPE and its absorption by the venous sinuses, as well as the fact that hydrocephalus can form if any part of the circulatory flow is disrupted [14]. Altered clearance of cerebrospinal fluid and increased intracranial pressure can also lead to hydrocephalus [15].

Existing studies have found that brain parenchyma can be permeable to water through water channel proteins and ion channels [16], which suggests that the cause of hydrocephalus may also be due to the accumulation of hyperosmotic substances and impaired transport of substances in the CSF. So, we found that osmotic pressure could be used to explain some phenomenon [16]. The central idea is the impairment of the maintenance of osmotic gradient homeostasis, where the osmotic gradient acts as a driving force for the transfer of water molecules, and the higher the osmotic pressure of the CSF within the ventricles, the more water collects in the ventricles [17], which in turn forms hydrocephalus [18].

The application of these theories can solve part of the problems in clinical treatment but not all of them, indicating that these theories have some limitations. Thus, we go to explore the relationship between CPE cells and hydrocephalus, and provide new targets for clinical treatment.

CPE have a relatively simple structure. They consist of a single layer of cuboidal epithelial cells that reside on a basement membrane. Beneath the basement membrane is a network of fenestrated capillaries that are surrounded by connective tissue composed of fibroblasts and immune cells [19]. By virtue of the tight junctions between them [20], a blood–CSF barrier is formed [21]. Faivre in 1854 and Cushing in 1914 were the first to suggest that the CPE secretes CSF, and De Rougemont et al. in 1960 found the first experimental evidence that the CPE secretes CSF [22]. Recent studies estimate that approximately 80% of the CSF is secreted by the CPE, with the remaining 20% coming from the brain interstitial fluid (BIF) [23]. The CPE is a very efficient secretory epithelial tissue in the body, secreting CSF at a rate of up to 0.4 ml/minute per gram of tissue [19]. The amount of CSF in the entire ventricular system of the human brain is estimated to be about 150 ml, but the CPE produces 500 to 600 ml of CSF every 24 h. This would suggest that the human body replaces CSF three to four times per day [24]. With such rapid secretion of CSF, if there is a blockage in the circulatory pathway [25], no matter which part is disrupted, CSF will accumulate rapidly and lead to ventricular dilation and even increased intracranial pressure, eventually leading to hydrocephalus [26]. Of course, this cause is only one of the causes of one type of hydrocephalus, which is obstructive hydrocephalus. Communicating hydrocephalus, on the other hand, is relatively uncommon due to an obstruction of CSF circulation, but rather an obstruction of CSF absorption. However, a new classification has also been proposed, which assumes that all hydrocephalus is obstructive except for those caused by excessive CSF production, but only the site of obstruction is different. This classification method has its validity.

In short, either intrinsic genetic factors or external environmental factors [12] that substantially promote the secretion of CSF from the CPE or disrupt the reabsorption of CSF [27] can lead to the accumulation of CSF in the ventricles and the development of hydrocephalus [28]. In addition, according to the new hypothesis, changes in osmotic pressure between CPE and capillaries can also lead to abnormal accumulation of CSF, which in turn leads to hydrocephalus. This is the relationship between the CPE and hydrocephalus, and the theory behind choroid plexus cautery. A more detailed description about this surgery will be developed below.

Whether it is based on the classical hypothesis of cerebrospinal fluid production or a newly proposed hypothesis, the current literature lacks a review of the relationship between the CPE and hydrocephalus formation. Understanding the relationship between the CPE and hydrocephalus is more conducive to our exploration of the pathogenesis of hydrocephalus and thus to the treatment of hydrocephalus.

## Normal physiological function of the CPE

### Brief description of the CPE

**Fig. 1:**
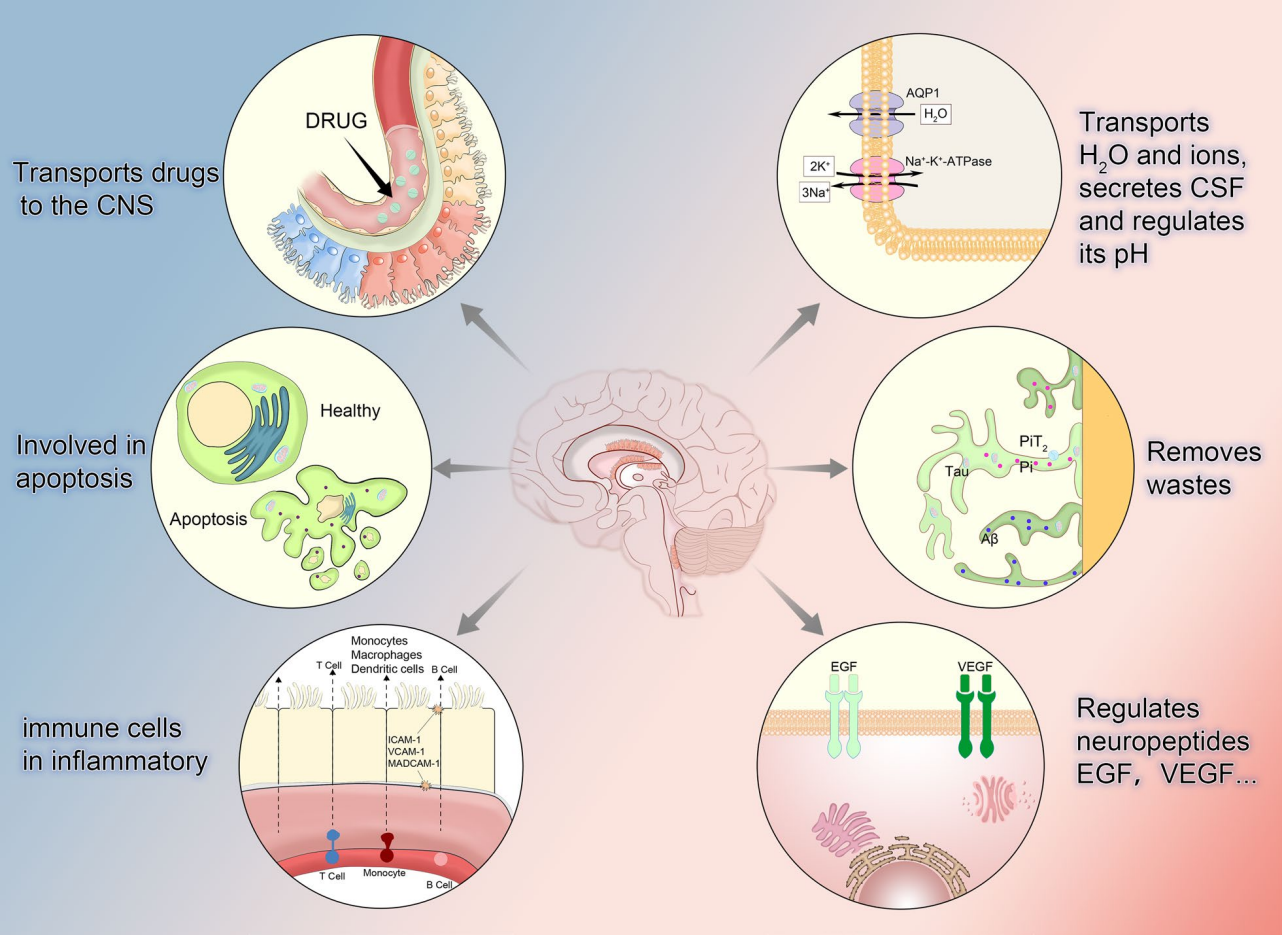
Physiology of choroid plexus epithelium. It transports water and ions, which are the major components of cerebrospinal fluid, and regulates its pH. These transport processes require the participation of transport proteins, such as aquaporin, which is involved in water transportation, and Na^+^-K^+^-ATPase, which is involved in the transport of various ions (mainly Na^+^ and K^+^). It also removes Pi through the protein PiT2, as well as other metabolic wastes. It is involved in the secretion and regulation of some neuropeptides. Serves as a transport channel for the delivery of certain drugs to the central nervous system. It is involved in the apoptosis pathways of other cells. During an inflammatory response, the connections between cells change, allowing some immune cells to pass through and participate in the response. CSF (cerebrospinal fluid), AQP (aquaporin), PIT2 (Pi transporter2), EGF (epidermal growth factor), VEGF (vascular endothelial growth factor), CNS (central nervous system), ICAM-1 (intercellular adhesion molecule-1), VCAM-1 (vascular cell adhesion molecule-1), Pi (inorganic phosphate)

In the ventricles of the human brain, there are four CPE located in the bilateral lateral ventricles, the third ventricle and the fourth ventricle. Together, they form the main component of BCSFB. During early embryonic development (fourth week) [29], cells originating from the ventricular canal, a special cuboidal epithelium [30], are extensions of the ventricles. Of these, the CPE in the fourth ventricle is the first to develop, at approximately ninth week. Then comes the CP in the two lateral ventricles and finally the CP in the third ventricle [31]. Researchers had found that canonical Wnt signaling is required in a precise and regulated manner for normal CPE development in the mammalian brain [32]. After the structure of the CPE is formed, its function is also fully developed soon [33]. The cells that make up the CPE are single layer cuboidal cells [34] and low cylindrical epithelial cells located in the basement membrane [35]. These cells can be observed under electron microscopy to contain a large number of mitochondria, a central nucleus, abundant Golgi apparatus, and vesicles that increase in size toward the surface of the lumen [36]. The large number of distributed microvilli results in a substantial increase in the surface area of the luminal membrane. Not only that, the originally flat base and side surfaces will appear like a maze of folds and wrinkles at the transition, which will also make the surface area increase. In the current state of the art cognitive understanding, researchers believe that in addition to microvilli, CPE cells must contain a small tufts of motile cilia [37]. Connections between CPE cells include tight junctions, intermediate junctions, and desmosomes [38], very similar to the proximal tubule of the kidney, both allow large amounts of fluid movement [39]. It is the structural basis of the CPE described above that determines its function of secretion of CSF and forms the theoretical basis for our study of its important role in the formation of hydrocephalus. Therefore, it is necessary to describe its structure briefly (Fig. 1).

### Functions of the CPE

#### The atypical polarity of the CPE

The inner and outer surfaces of multicellular organisms are covered with epithelium, which keeps out most invasive substances. In addition, there are a number of important functions, as described below. And all these functions are based on the same basis of cell biology. In order to regulate selective transport processes to maintain a proper internal environment, membrane proteins appear asymmetrically distributed. This requires the expression of specific transport proteins by cells according to their functional needs and the proper sorting and transport of these membrane proteins. This is known as cell polarization [40]. The CPE is polarized in a similar way to other epithelia, with a basal membrane, separated from the luminal membrane by means of tight junctions, which are lined with microvilli and motile cilia. However, CPE have a distinctive atypical feature which is called atypical polarization. This is manifested by the expression of proteins such as Na^+^-K^+^-ATPase, sodium potassium chloride co-transporter 1 (NKCC1) [41] and Na/H exchanger 1 (NHE1) in the luminal membrane, which are supposed to be located in the basal membrane, while proteins such as anion exchanger 2 (AE2) remain in the same position [42]. These proteins are incorporated into specific amino acid sequences during endoplasmic reticulum synthesis, which in turn are recognized by the rough endoplasmic reticulum–Golgi complex axis, which determines the classification of proteins for targeted transport vesicles in the trans-Golgi complex network [43].

#### CPE transports water and ions and secretes CSF

The CPE is composed of a single layer of cuboidal epithelial cells whose most important function is to produce CSF. In order to secrete CSF, it is mediated by a variety of membrane proteins that allow water and solutes to be transported across the epithelium, across the BCSFB, and from the blood side to the CSF side. The relevant biophysical principles have not yet been unified regarding the study of this situation, and among many relevant opinions, isotonic transport is currently widely recognized and studied with emphasis. To explain how water transport in the CPE is coupled with solute transport, resulting in near-isotonic transport, several models have been proposed to study the molecular mechanisms and have been continuously refined. Among these are three-compartment model [44], standing gradient model and its modifications, osmotically driven water transport model [45], water [46] and ion cotransport [47], and tight junction-based models [48]. In the following, the descriptions are classified according to the type of substance being transported.

Water transport in the CPE is associated with AQP, a transmembrane water transport protein. AQP1, AQP3, AQP4 [49], AQP5, AQP8, and AQP9 are currently identified in the brain [50], with AQP1 being the most critical and playing an important role in the overall process of water transport [51]. AQP1 and AQP5 are key water channel proteins that control water flux within the CP barrier and the production of CSF [52]. In mature tissues, AQP1 is mostly expressed apically in the epithelium, indicating solutes diffusion from the CPE to the ventricles [53]. In contrast, AQP1 expression is relatively absent in the basement membrane, suggesting a possible effect of paracellular water transport [19]. Most of the current studies are based on animal models, and few experiments have been done in humans. In animal models, AQP1 acts as a regulator of CSF production with adaptive and protective effects, but further studies are needed to demonstrate whether this link exists in humans. Differential changes in AQP1 expression may be associated with different types of hydrocephalus [54]. After intraventricular hemorrhage (IVH) combined with post-hemorrhagic ventricular dilatation (PHVD), AQP1 expression rises first as an early adaptive mechanism to increase intracerebroventricular water content and decrease CSF osmolarity [52]; it decreases later, belonging to autocrine regulation, decreasing water permeability in the CP and decreasing CSF production. The molecular mechanism behind this is unclear. The expression of AQP5 rises, contributing to the protection of the extracellular matrix and the strength of tight junctions, thereby strengthening the function BCSFB. In obstructive hydrocephalus and subarachnoid hemorrhage (SAH), the expression of both AQP1 and AQP4 rises, probably to speed up water transport, allowing an increase in the water content of the CSF, thus decreasing the osmotic pressure [55]. In communicating hydrocephalus, AQP1 expression is also elevated [56].

Na^+^ is the most important ion that powers the secretion of CSF. The most important of these transporter proteins is Na^+^–K^+^-ATPase, which, in addition to transporting Na^+^ into the CSF, forms electrochemical gradients to help other ion transporters [57]. The epithelial Na^+^ channel (ENaC) is a channel for Na^+^ to enter via the basolateral membrane [58]. The NKCC1 is located mainly in the basolateral membrane and to a lesser extent in the luminal membrane. Although it is abundantly expressed, its practical significance remains to be determined [59]. The electrogenic co-transporter NBCe2 (or NBC4) is Na^+^–HCO_3_^−^ cotransporter. It is located in the luminal membrane and in addition to transporting Na^+^ according to the ratio of one to three of Na^+^ and HCO_3_^−^ [60], and it also regulates the pH of the CSF with the help of transporting HCO_3_^−^ [61]. NHE1 is located in the luminal membrane and is responsible for maintaining the pH of the CSF rather than directly promoting its secretion [62]. Na^+^–borate cotransporter can transport Na^+^ and borate into cells, but its specific mechanism of action is not known.

The mechanism of K^+^ transport by the CP is very complex and relies on transport proteins in addition to paracellular pathways. K^+^ channels in the luminal membrane recirculate K^+^ [63], which creates a negative membrane potential and thus maintains a certain electrochemical gradient in the CPE [64], and also maintains the activity of Na^+^–K^+^-ATPase [65] and NKCC1 that transport K^+^ from the CSF to the CPE. KCNQ1 and KCNE2, expressed in the luminal membrane, are also involved in the formation of K^+^ channels [66]. The K^+^–Cl^−^ cotransporters (KCC), expressed in the basolateral membrane as KCC3a and in the luminal membrane as KCC4 [67], their exact contribution needs to be further evaluated using gene knockout method [68].

Ca^2+^ plays many important roles within the central nervous system, regulating many physiological processes and is a key factor in maintaining the stability of various signaling pathways [69]. It has been shown that the transportation of Ca^2+^ in CPE is involved in transporter proteins and not by simple diffusion through paracellular pathway [70]. There is currently a theoretically feasible model in which Ca^2+^ is taken into cells by transient receptor potential vanilloid 5 (TRPV5) and transient receptor potential vanilloid 6 (TRPV6) from the basement membrane, bound to calcium-binding proteins, transferred to the luminal membrane, and released into the CSF via Ca^2+^-ATPase (PMCA) and Na^+^/Ca^2+^ exchanger (NCX), but this model lacks experimental evidence [71]. 5-HT_2c_ receptor agonists can promote spontaneous calcium activity [72]. GSK1016790A-specific agonists activate transient receptor potential vanilloid 4 (TRPV4) located in the luminal membrane, which in turn leads to increased ion flux and conductance. TRPV4 is a central protein that controls CSF production through stimulation of multiple effectors. It is also a non-specific cation channel that allows Ca^2+^ to flow into cells and stimulates calcium-sensitive potassium in a tissue-specific manner channels that can be inhibited by two different specific antagonists, which are named HC067047 and RN1734 [73]. The Slc29a4 gene regulates the expression of plasma membrane monoamine transporter protein (PMAT), a multispecific organic cation transporter protein located in the luminal membrane and its transportation is independent of Na^+^. It serves to protect the brain from cationic neurotoxins by removing the corresponding substances from the CSF [74]. The transport of anions by the CPE is closely related to Cl^−^ and HCO_3_^−^, and trans-cellular transport for Cl^−^has been demonstrated in several experiments [75]. Therefore, some drugs inhibit the production of CSF by inhibiting the excretion of Cl^−^. Just for example, arginine pressor (AVP) activates receptor V_1_, which inhibits the ability of the CPE to transport Cl^−^, thereby reducing CSF formation [76]. AE2, located in the basement membrane, mediates Cl^−^ efflux and HCO_3_^−^ uptake, and the gene encoding it belongs to the Slc4 family. The Slc4a10 gene, also in the Slc4 family, encodes an Na^+^-dependent Cl^−^/HCO_3_^−^ exchanger (NCBE) involved in Cl^−^ and HCO_3_^−^ transport, and CPE lacking NCBE appears to omit molecular mechanisms involving CSF secretion rather than compensating for the loss of sodium load. The reason that knocking out this gene is more harmful than knocking out the AQP gene is that the former contains more than one signaling pathway [77]. The lack of NCBE seems to make the transport program of epithelial cells tend to be dormant [78]. In addition to the Cl^−^ channels and HCO_3_^−^ channels located in the luminal membrane, which are responsible for the transport of Cl^−^ and HCO_3_^−^, the NKCC1 and the KCC are also involved [19].

In addition to the transport of water and ions, the CPE has been shown to be involved in the transport of albumin and some amino acids. The soluble and cell surface albumin-binding glycoproteins secreted protein acidic and rich in cysteine (SPARC) is thought to be involved in the transport of albumin into the brain and is the docking site for albumin, mediating the uptake of albumin from the blood by the CPE, crossing the blood–CSF barrier from the basement membrane and transferring it to the CSF [79]. Sodium-dependent amino acids co-transporter, system A member (SNAT1) actively transports glutamine from the blood to the CPE and then releases it into the CSF via sodium-dependent amino acids/H^+^ co-transporter (SNAT3) and large neutral amino acid transporter type 2 (LAT2). To maintain the amino acid concentration gradient between plasma and CSF, the CPE drives CSF reuptake of essential amino acid substrates for this retrotransport protein via efflux of LAT2. However, other amino acid transporter proteins have only been detected at the level of mRNA and have not been localized at the protein level [80]. The atypical polarization characteristics of the CPE underlies its specific function. With the help of transport proteins in the membrane, the CPE is able to transport water, ions and other substances across the cells, across the BCSFB and into the CSF from the blood, thus serving to secrete CSF. Among the many ions, the secretion of CSF by the CPE depends mainly on the transepithelial cellular movement of Na^+^, Cl^−^, and HCO_3_^−^ [81].

To study the problems related to CSF clearly, it is necessary to study these transporter proteins first. As far as we can see from the review, some of the transporter proteins have been discovered and their role were confirmed, others have only been discovered but their role has not yet been confirmed, and we presume that there are still some that have not been discovered [19]. Among these identified transport proteins, the specific molecular mechanisms are still unclear, and models are still being developed and updated. To some extent, these transport proteins can be used as therapeutic targets for related brain diseases such as hydrocephalus. In fact, most of the drugs currently used to treat hydrocephalus work by inhibiting the production of cerebrospinal fluid by inhibiting these transport proteins or their related enzymes. However, these drugs are not effective enough, so we need to study these transport proteins more thoroughly and develop effective drugs according to their mechanism of action. Therefore, the study of these proteins and the molecular mechanisms behind them is widely promising.

#### Removes metabolite products

The concentration of inorganic phosphorus in the CSF is lower than in plasma, and how and why it is controlled is not known. However, some studies have found that Pi transporter2 (PIT2) in the CPE may be part of the phosphorus sensory or regulatory pathway. The active clearance of phosphatidylinositol from the CSF has been linked to the sodium-dependent transporter protein PIT2 [82]. Proteins such as uric acid transporter protein (URAT1) located in the basolateral membrane and breast cancer resistance protein (BCRP) located in the luminal membrane are involved in the transport of the uric acid, but the specific physiological as well as pathological functions need to be further investigated [83]. With increasing age, the expression of β-amyloid (Aβ) clearance-related proteins in CPE tissues decreases and the clearance capacity decreases, causing the accumulation of Aβ [84]. Representative among these clearance proteins are low-density lipoprotein receptor-related proteins (LRP-1 and LRP-2), P-glycoprotein (P-gp), and receptor for advanced glycosylation end-products protein (RAGE) [85]. Among them, LRP-1 and P-gp are efflux transporters [86], and LRP-2 and RAGE are responsible for circulating Aβ from the periphery to the brain [87]. These proteins are thought to promote Aβ clearance synergistically or complementarily with insulin-degrading enzymes (IDE) [88], and sex hormones may also play a role in this process [89]. Impaired clearance of metabolic products, whether they are needed by the body or waste products, may cause changes in the osmotic pressure of the CSF, which in turn may lead to accumulation of CSF and cause hydrocephalus. Therefore, focusing on the metabolite clearance pathways involved in the CPE may be a target for subsequent studies.

#### Involved in immune modulation

Leukocytosis in the CSF is widely used clinically to assess inflammatory conditions in the central nervous system. This suggests that the CP, located at the junction of blood and CSF, is a multifunctional organ that serves as a gateway for immune cells to enter the CSF and plays an important role in immune surveillance and regulation of immune cell transport. However, studies on the principles of BCSFB control of immune cells are limited, and only some features of the CPE have been identified [90]. Epithelial cells secrete adhesion molecules and chemokines that control the selection of metastatic cells [91]. The rapid flow of blood in the CP provides excellent environmental conditions for immune cell circulation [92]. Studies have demonstrated that the CPE cell surface expresses adhesion molecules and presents antigens to T cells. The expression of ICAM-1 (intercellular adhesion molecule-1), VCAM-1 (vascular cell adhesion molecule-1) and P-selectin may demonstrate that the CP has an environment that supports immune cell trafficking and immune surveillance [93]. Epithelial cells release chemokines in a polarized manner that attract immune cells to the surface and promote migration [94]. This migration, however, is via a paracellular pathway and does not disrupt the restrictive barrier at the epithelial interface [95]. These suggest that epithelial cells are a key factor in immune cell transit. After inflammatory stimulation, the TLRs signaling pathway is activated, leading to iNOS expression via NFκB [96]. The CP as a niche for T cell stimulation [97], contains CD4^+^ effector memory cells that retain a TCR (T cell receptor) profile against CNS antigens. T cell-mediated immunity affects the CP and attenuates the secretion of IL-4 (interleukin 4) [98], which has been shown to be associated with brain aging and may serve as a therapeutic target for reversing or halting age-related diseases such as dementia [99].

#### Regulation of CSF pH

Controlling the pH of the CSF is very important because it directly affects the pH of the BIF closest to the ventricular system, which in turn causes the onset of certain diseases [100]. It was initially puzzling that CSF, which contains only a very small amounts of protein-buffering substances [101], was able to keep its own pH stable within a narrow range in the face of acid–base changes. For this reason, several researchers have proposed the idea that CPE cells are involved in pH regulation of the CSF, and many acid–base transporter proteins that may be involved in pH regulation of the CSF have been identified in CPE cells and localized by immunohistochemical analysis. The NCBE on the outer side of the basement membrane is a Na^+^-dependent Cl^−^/HCO_3_^−^ exchanger that, together with HCO_3_^−^, absorbs Na^+^ from the blood side [102]. The anion exchanger AE2 crosses the basolateral membrane to discharge HCO_3_^−^ [103]. NBCe2, located on the luminal membrane, is a Na^+^/HCO_3_^−^ co-transporter and exports Na^+^ and HCO_3_^−^ to the CSF in a one-to-three ratio at high CO_2_ concentrations [104]. The NHE1 and NHE6 are both Na^+^/H^+^ exchangers, importing Na^+^ into cells and exporting H^+^ into the CSF. NHE6 is located in the structural domain of the luminal membrane and regulates pH in concert with V-ATPase. The CLC-3, CLC-4, CLC-5, and CLC-7 are all Cl^−^/H^+^ exchangers that export Cl^−^ outward. In addition to this, there is V-ATP [105] involved in regulating pH, but its effect is not significant. Most of the V-ATP is located in small intracellular vesicles, with only a small fraction in the luminal microvilli region; this fraction does not promote acid secretion in the presence of high extracellular pH, but can be activated by cAMP in a transport-independent manner.

#### Regulates the neuropeptides to help recover from damage

In the CSF secreted by CPE, neuropeptides are regulated by an endocrine-like mechanism that uses the bulk flow of CSF to aggregate neuropeptides, including brain-derived neurotrophic factor (BDNF), epidermal growth factor (EGF), fibroblast growth factor (FGF), glial cell line-derived neurotrophic factor (GDNF), insulin-like growth factor (IGF), nerve growth factor (NGF), pituitary adenylate cyclase activating polypeptide (PACAP) and vascular endothelial growth factor (VEGF) [106]. They are transported to the damaged part near the target cells to help repair the brain damage caused by traumatic brain injury. If appropriate growth factors or neurotrophins are used to achieve pharmacological facilitation of the CSF, it is possible to help regulate neuropeptides that may improve the nervous tracts within CNS and thus help restore the injury.

#### Helps in the clearance of the infection with responses of the CPE during infection of the CNS

The tightly connected CPE is involved in constituting the BCSFB [107], which is one of the major barriers of the CNS [108]. It allows the exclusion of pathogens capable of causing disease [109]. However, certain pathogens contain a range of pathogenic factors that can cross the barrier [110] and other defense mechanisms and cause disease [111].

The CPE is a highly vascularized tissue that provides an important interface between the blood and the CNS [112]. However, some pathogens can break through the physical barriers, biochemical barriers [113] and immune barriers [114] by various means, which turns the CPE into a portal of entry for pathogens into the CNS. Pathogens interact with host cells and stimulate specific cellular signal transduction. The main manifestation is the cell surface Toll-like receptors (TLR family) [115], which activate transcription factors (TF), commonly NF-κB [116], mainly with the help of phosphorylation. In turn, they regulate cell death, both apoptosis and necrosis. At the same time, pathogens also regulate the gene expression profile of the host cell, both up- and down-regulated, as evidenced by altered secretion profiles of some proteins. There are surface protein receptors and matrix metalloproteinases involved in the inflammatory response, as well as cytokines and chemokines that attract immune cells to clear pathogens. This plays a role in helping to clear inflammation.

#### Regulating the apoptosis pathways and the Aβ-induced damage.

The p75 neurotrophin receptor (p75NTR) is expressed in the CP and is localized to the luminal membrane of epithelial cells. In neurons, p75NTR receptor has a dual function: promoting survival together with TrkA in response to NGF, and inducing apoptotic signaling through p75NTR. which in turn alters antibody clearance and inhibits neuronal growth by inducing apoptosis through p75NTR. The role of p75NTR in antibody-induced cell death was confirmed by blocking the biological activity of p75NTR [117].

#### Transport of drugs in the brain

The CP, a component of the blood–CSF barrier interface, is an epithelium that can be used to sparingly deliver drugs to the brain. Drugs delivered from the blood to the lateral ventricles are delivered through the CSF to targets within the ventricles and are used to regulate secretion and help treat disease [118]. The underlying principle that enables us to develop drugs for distribution to brain targets along the CSF pathway is that the CP delivers neurotrophic and growth factors to neurons by means of the CSF network. A large number of microvilli are distributed in the luminal membrane, providing a large surface area for molecular fluxes, and a large number of mitochondria provide energy for secretion and reabsorption. The ultrastructural features of these organelles reveal the cellular mechanisms behind them. The constant movement of water molecules carries polar molecules, which drive the transport of drugs in the CSF. The search for solutions with minimal side effects and toxicity has led to innovative explorations by a large number of researchers [119], including bone marrow cell infusions [120], CP transplantation [121]. Some researchers have found through experiments that receptors for AVP (arginine vasopressin) and ANP (atrial natriuretic peptide) [122] in CP are targets for agents to reduce CSF formation, which can be used to treat hydrocephalus.

### Abnormalities of the CPE

#### Cilia alteration due to gene deletion or hydraldehyde treatment

Cilia (kinocilia) play an important role in the normal physiological regulation of the CPE, and treatment with hydantoin or Tg737^orpk^ mutants can lead to cilia dysfunction, which in turn interferes with signaling pathways, disrupts CSF flow, resulting in increased transcytosis activity in the CPE, increasing CSF secretion and decreasing its pH. Ciliary dysfunction leads to increased cAMP content and activation of PKA, which allows phosphorylation of extracellular signal-regulated kinases (ERKs), which in turn regulates related ion channels and ion transport proteins, resulting in increased secretion of Cl^−^ and HCO_3_^−^, ultimately manifesting as increased CSF secretion [27].

#### Promotes inflammatory response after injury

**Fig. 2:**
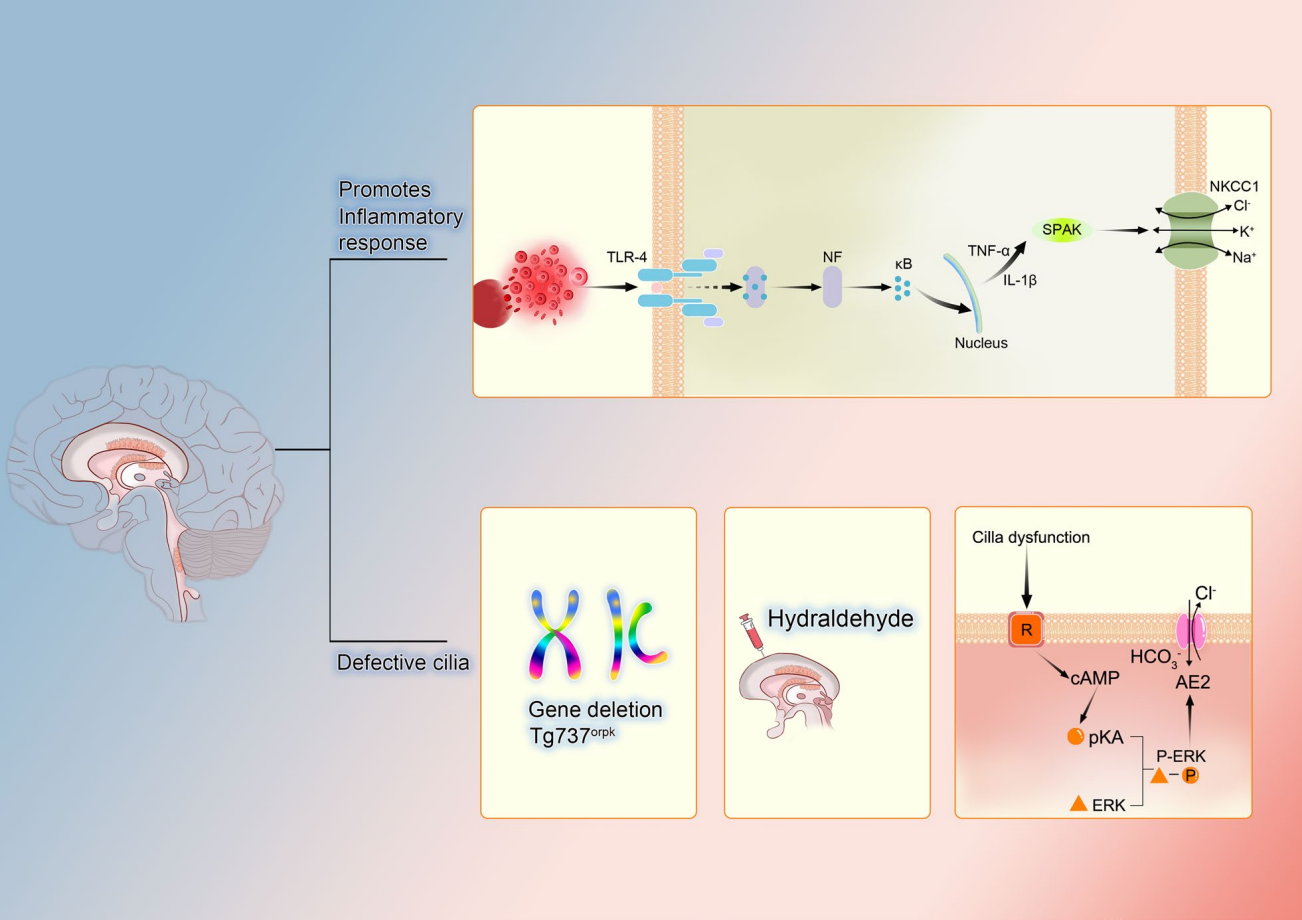
Pathology of choroid plexus epithelium. The pathological process of choroid plexus has two main pathways. One promotes inflammation by acting on transporter NKCC1 with the help of TLR4. And NF-κB will be separated into NF and κB, the latter acts on SPAK through pro-inflammatory factors such as TNF-α and IL-1β. The other is the abnormal condition of cilia, mainly caused by gene mutation and hydraldehyde. Cilla dysfunction acts on the receptor, binds the pKA and ERK through the action of the second messenger cAMP, acts on the transporter AE2 and upregulates its function. TLR4 (Toll-like receptors 4), NKCC1 (Na–K–2Cl co-transporter 1), AE2 (anion exchanger2),

IVH causes the rupture of blood vessels, leading to a hemolytic phenomenon that releases extracellular hemoglobin (Hb) into the CSF and activates an inflammatory response [123]. IVH stimulates the TLR4/NF-κB signaling pathway, activating the Ste20-type stress kinase Spak located in the luminal membrane, which binds to NKCC1 to generate a complex that increases ion transport and increases CSF secretion [124]. By virtue of the JNK signaling pathway, AVP promotes the synthesis of pro-inflammatory mediators [125], which include TNF-α and IL-1β, and can inhibit the effect of TRPV4 on the permeability of ion transport across epithelial cells [126]. These pro-inflammatory mediators can also be secreted by the CPE because of spinal cord injury, but the exact mechanisms involved have not been well studied [127]. In addition to these pro-inflammatory mediators, traumatic brain injury (TBI) promotes the synthesis of the monocyte chemotactic substance CCL2, which is released into the CSF at the luminal membrane as well as at the basolateral membrane and promotes leukocyte crossing of the epithelial barrier [128] (Fig. 2).

### Hydrocephalus caused by the abnormalities of the CPE

#### Based on the circulation theory

CSF is produced by the epithelium of the CP, travels through a certain pathway, and is finally absorbed by the venous sinuses. Injury to any part of this process can lead to abnormal CSF levels and result in hydrocephalus.

The inflammatory response induces an increase in CSF secretion by the CPE to affect directly, or induces ciliary dysfunction to affect indirectly. In the animal model of microbial infection, the CP exhibited distinct histological features altered by focal leukocyte infiltration and lamellar loss of epithelial cells, in addition to the reduction of epithelial cell microvilli observed by electron microscopy [129]. Obesity may also act as an inflammatory state, allowing increased cytokines in the CSF, leading to fibrotic changes that are arachnoid villi obstruction and reduced CSF uptake [130]. CSF circulatory dynamics are impaired, and although problems can occur at any point in the circulatory process, the vast majority of studies have focused on cilia dysfunction. Lack of functional transcription factor foxJ1 leads to cilia deficiency, resulting in impaired circulatory kinetics [131]. The absence of cilia increases the cAMP content in the CSF, which activates PKA and allows phosphorylation of ERKs, increasing the secretion of CSF. It was observed that the ciliated CPE had higher trans-cellular activity, which may have increased CSF secretion along the trans-cellular secretory pathway of the AQP1 street, or it may have increased trans-cellular transport of proteins, which increased CSF osmolarity and acted as a driving force to drive the AQP-mediated fluid transport [132]. Impairment in the formation of the CPE can also lead to a series of subsequent problems, for example, the functional transcription factor E2F5 plays a role in the maturation of the CPE, although the exact role is not yet precisely understood [131]. In the rat model of experimental hydrocephalus, with the help of transmission electron microscopy, changes in cellular characteristics such as cell membrane fragmentation, large numbers of primary and secondary lysosomes, vacuoles, and enlarged cell gaps could be observed [133]. This suggests that any incomplete immaturity of CPE cells can affect CSF secretion and lead to hydrocephalus.

Impaired transport of water molecules and ions is the most important cause of abnormal CSF secretion, which will be described specifically below.

#### Based on the osmotic pressure theory

The accumulation of hypertonic substances in the CSF alters the osmotic pressure of the CSF and affects the transport of substances.

The main and most important cause is a series of transporter proteins for water molecules and ions, either missing or malfunctioning, or in the wrong place, which may cause abnormal secretion. The functional transcription factor p73 regulates the production and reabsorption of CSF by regulating the water channel protein AQP3. This then leads to non-obstructive hydrocephalus [131]. When intracranial pressure rises for some reason, the brain responds to the situation by reducing CSF, and the molecular mechanism behind this is the reduced ability of Cl^−^ channels to release Cl^−^ [134]. Higher level of estrogen increases the levels of cortisol and TNF-α, which in turn affects a number of transporter proteins, including the water channel protein AQP4, causing abnormal secretion of CSF [130]. In addition to these examples, there are some unknown conditions that lead to other transporter proteins that are impaired in transporting Na^+^, Cl^−^, K^+^, and HCO_3_^−^ ions, all of which could be the molecular basis for the generation of hydrocephalus.

The presence of large amounts of hypertonic substances in the CSF will produce a higher osmotic pressure that acts as a driving force to affect water transport. In thrombin-derived hydrocephalus, part of the cause can be attributed to the degradation of the blood–CSF barrier by VE-cadherin through activation of signaling pathways such as PAR1/p-SRC/p-PAK1, which increases its permeability and leads to leakage of CSF [135]. Toxic substances that are not removed from the CSF and accumulate in excess can damage the epithelial cells of the CP and are a possible cause of hydrocephalus, typically hemoglobin and free iron.

**Fig. 3:**
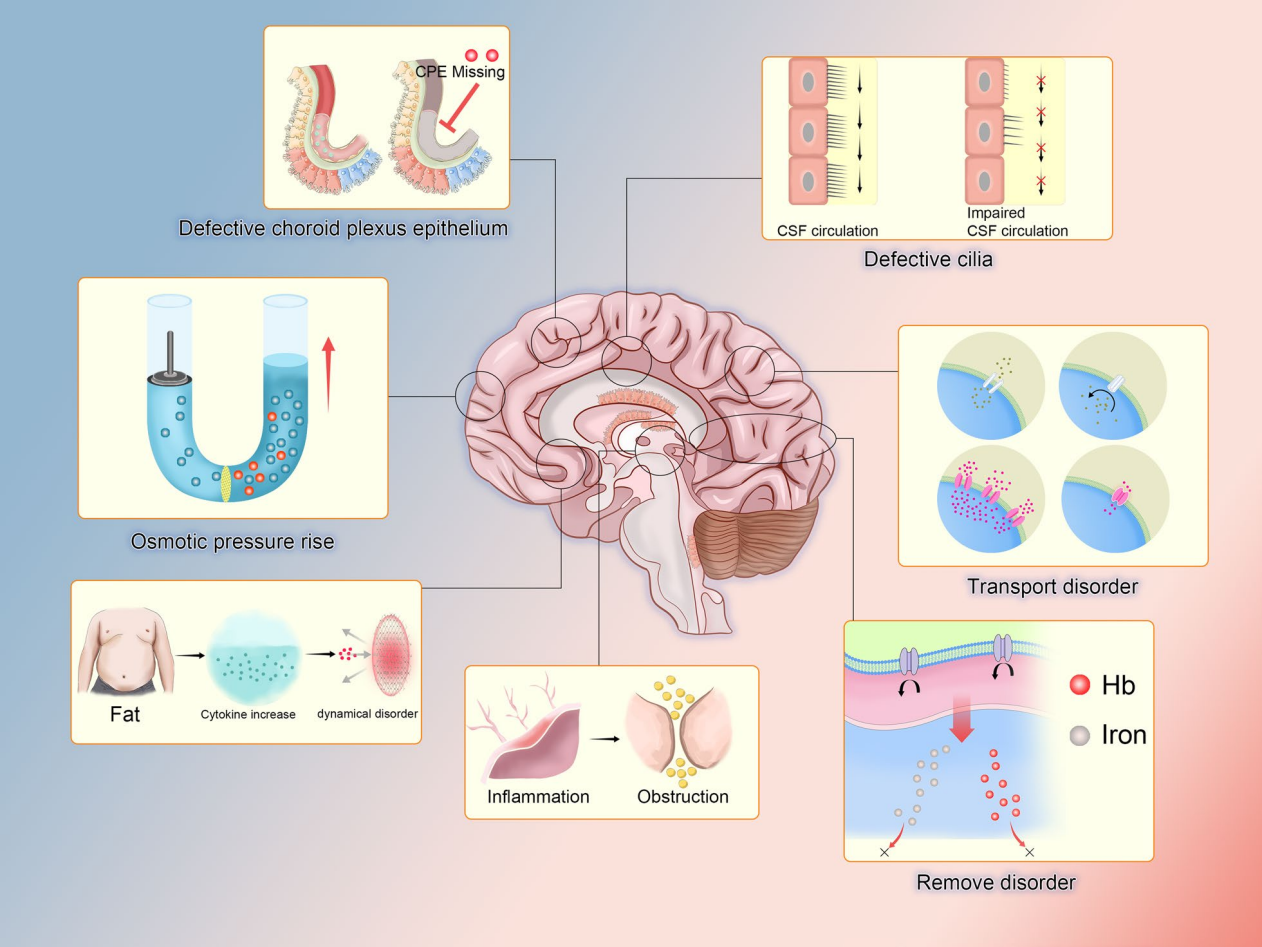
Hydrocephalus related to choroid plexus epithelium. There are many lesions of choroid plexus that can lead to hydrocephalus. Defective cilia, whether short, sparse or missing, can cause obstruction of cerebrospinal fluid flow. The disorder of transporters, both the decline of transport capacity and the decrease of protein quantity, will cause problems in cerebrospinal fluid secretion. The impaired ability of efflux transporters to remove metabolic waste will increase osmotic pressure and affect secretion. Obstruction caused by inflammation is also the reason of obstruction of cerebrospinal fluid secretion. The increase of cytokines caused by obesity leads to the obstacle of reflux. Increase of cerebrospinal fluid osmotic pressure and absence of choroid plexus epithelial cells are both the reasons of hydrocephalus. Hb (hemoglobin), CPE (choroid plexus epithelial)

In conclusion, whether the process of hydrocephalus formation is understood with the help of the circulatory theory or the osmolarity theory, any disruption of either of these components may be a factor in the pathogenesis, the difference only lies in whether it is common and whether the underlying molecular mechanisms are understood. Only some of the possible etiologies have been mentioned above, and in an abbreviated manner, without addressing too many of the deeper mechanisms. Researchers are still required to speculate boldly and seek proof carefully (Fig. 3).

### Possible treatment options based on CPE

There are neither drugs that are exclusively effective in treating hydrocephalus, nor is there a surgical procedure that can solve the problem once and for all. The lack of in-depth basic research on hydrocephalus [136] and the lack of clarity on the true molecular mechanism are the fundamental reasons why we cannot come up with an efficient treatment [137]. Although the research is not thorough enough, there are still more ways to relieve or even cure hydrocephalus with the research of countless predecessors [124]. Of course, the mechanisms behind these methods are different, so here we will mainly talk about feasible treatments related to the CPE. The current treatment options based on the CPE are focused on three areas, namely direct surgical destruction of the CP, drug or other means to inhibit the transporter protein, and cell transplantation (still in the research stage).

Surgical destruction of the CP, CP cautery using the electric knife has been used clinically, and the results of the treatment, although not perfect, are still considerable. In contrast to traditional surgical methods, immunotoxin-mediated CP ablation [138] is now available and is also a possible treatment for hydrocephalus, but it has only been studied in rat models and is not yet eligible for clinical use. Moreover, this approach does not necessarily stop all CSF production, since nearly 80% of the CSF is secreted by the choroid plexuses, with the remaining 20% coming from the interstitial fluid of the brain which is generated by the BBB More importantly, the CP is not only an organ for CSF secretion, but it also has many other important physiological functions that, if disrupted, could cause irreparable and greater damage.

Although the CPE has many types of transporter proteins, only a few have been used as targets for the treatment of hydrocephalus, and almost all have been studied in animal models and have not been applied in human studies. A brief description of the relevant studies is presented.

The water channel protein AQP family is an important class of transporter proteins responsible for the transport of water molecules, and therefore studies using them as targets are widespread. Upregulation of AQP has been found in several types of hydrocephalus or other related diseases, for example, upregulation of AQP1 and AQP4 in SAH, suggesting that we may be able to inhibit CSF secretion and alleviate hydrocephalus by downregulating AQP symptoms. There may also be a mechanism for endocytosis of AQP1 in the CPE to slow down CSF secretion during hydrocephalus. Although the CSF production in mice with knockout AQP1 was reduced by only thirty percent, this was sufficient to affect the formation of hydrocephalus [139], suggesting that inhibition of AQP1 is a possible target.

IVH activates the STE20-type stress kinase SPAK via TLR4 [140], which binds to and phosphorylates the NKCC1 cotransport protein at the apical membrane [124]. Genetic deletion of TLR4 or SPAK was found to normalize CSF secretion, suggesting that inhibition of the TLR4-NFκB signaling pathway or the formation of the SPAK-NKCC1 complex could have a therapeutic effect on hydrocephalus. Bumetanide can also inhibit NKCC1 [24].

Na^+^–K^+^-ATPase is an important transporter protein responsible for the transport of Na^+^ and K^+^ and plays a very important role in the secretion of CSF. It has been found that ANP [141] binds to the CPE, producing cAMP, inhibiting the sodium pump and reducing sodium uptake, leading to altered ion transport and slowed CSF production. It also induces dark cell production, cell shrinkage, cytoplasmic condensation, and normal organelle appearance, possibly in an out-of-work state, associated with a decrease in CSF. Curcumin [142], the main component of turmeric rhizome, inhibits Na^+^–K^+^-ATPase and helps regulate CSF production. The use of ouabain inhibits Na^+^–K^+^-ATPase, leading to a decrease in CSF production [24].

The blood–brain barrier is the key issue, which limits the entry of therapeutic compounds or systemically transplanted cells into the brain. The intended application of cell therapy is to regenerate the disrupted VE and drug delivery to improve brain microcirculation and neurological function. CPE cell transplantation [143] for hydrocephalus is theoretically feasible, but still in the theoretical stage, still under investigation, more experimental results are needed to further determine the feasibility, and it is currently a hypothetical scenario.

The methods mentioned above, both those already applied and those still under investigation, are based on the theory of CSF circulation flow associated with the CPE. The newly proposed theory of CSF osmolarity, on the other hand, offers the possibility of new treatment options. Removal of substances accumulated in the ventricles, such as hemoglobin and its degradation products, platelets, and leukocytes, reduces osmotic pressure, attenuates damage to CPE cells, and decreases CSF secretion. Upregulation of efflux molecular transport proteins also removes substances accumulated within the ventricles and relieves hydrocephalus.

**Fig. 4:**
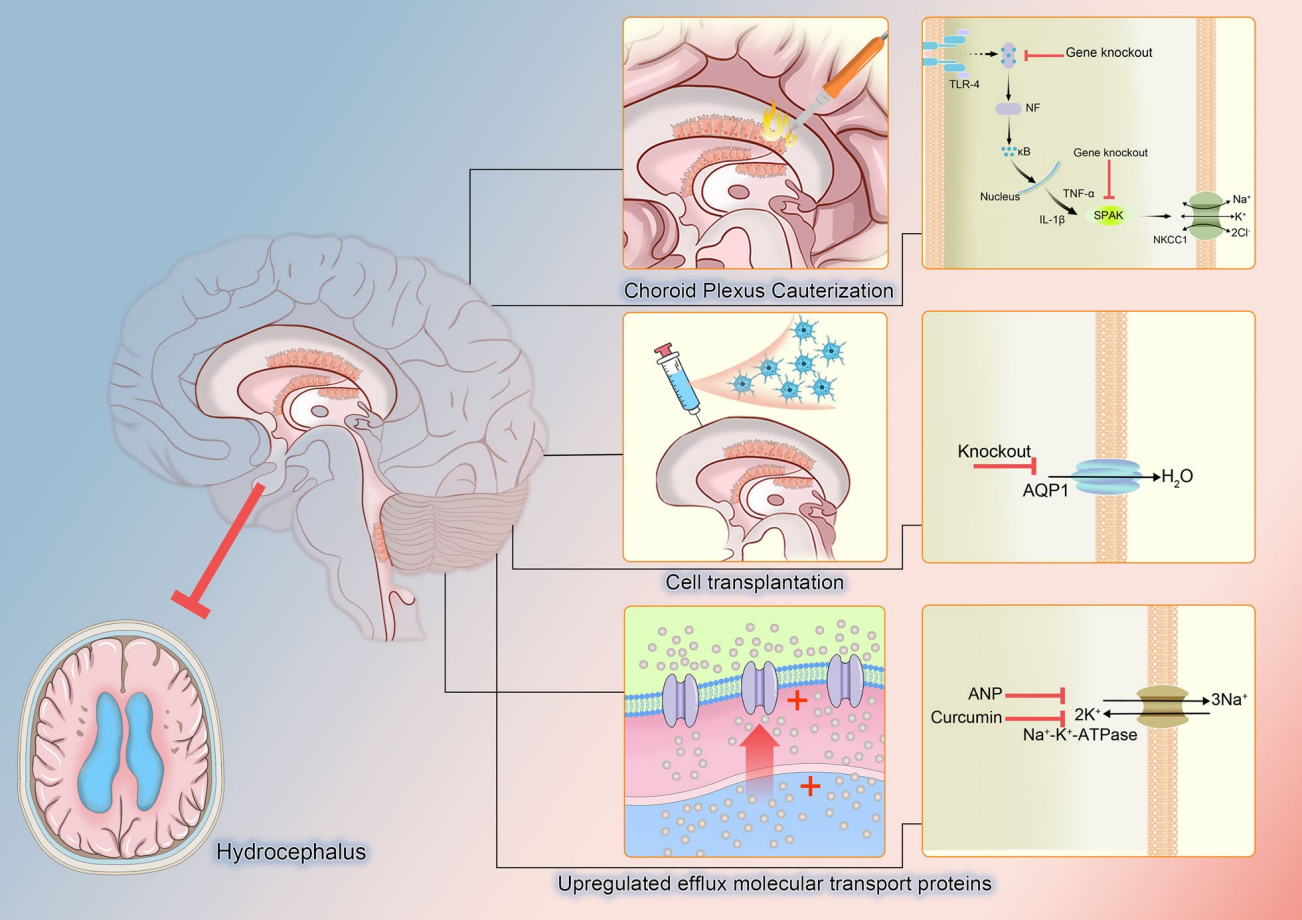
Therapy of hydrocephalus. At present, there are many methods that have been clinically applied or may be used as a target for the treatment of hydrocephalus. Choroid plexus cauterization is one of the important representatives of surgical methods. Cell transplantation of CPE is a research hotspot in the future. Upregulation of transporters is an effective way, both in transport capacity and protein quantity. In addition, using special drug blocking or gene knockout to inhibit inflammatory response pathway and some important transporters, such as AQP1 and Na^+^-K^+^-ATPase, is also a hot research direction

It is worth noting that these two theories are not completely separated, but are mutually corroborative. A certain causative factor or a certain treatment, interpreted from different perspectives, may belong to different theories, but in the final analysis, the two are essentially related. We need to continue our research to deepen our understanding of both theories in order to identify the correct and comprehensive theories to explain hydrocephalus and to provide new and reliable ideas for its treatment (Fig. 4).

## Conclusions and outlook

As a disease with diverse etiologies, complex conditions, and imperfect underlying theories, hydrocephalus requires more attention from researchers. The widely accepted theories are the CSF circulation flow theory and the osmolarity theory. These theories demonstrate the causative factors from different perspectives and are somewhat different, but not completely disconnected. The same pathogenic factors can be divided into different theories, which to some extent indicates that these theories interpenetrate and interdepend on each other, only that deeper research is needed to integrate them.

At present, the clinical treatment of hydrocephalus mainly uses some dehydrating drugs to reduce edema, and surgery is roughly divided into shunt surgery and ventriculostomy (such as endoscopic third ventriculostomy). Although they have been used clinically with some success, they are not considered to be specific and there should be a more focused approach to solve hydrocephalus efficiently.

This article provides a comprehensive overview of the role of the CP in hydrocephalus formation, and although some of the mechanisms remain unclear, it has provided new targets, with multiple transport proteins located in the parietal and luminal membranes of the CPE being a hot topic of study. In addition, it would be extremely valuable to clarify the unknown molecular mechanisms, both to deepen the understanding of the mechanisms of hydrocephalus formation and to provide new targets for possible therapeutic modalities.

In conclusion, the study of the CP, at any level, has its unique significance. Although studies have denied that the CP acts as a power pump in the circulatory flow of CSF, it is undeniable that the CP has a crucial role in the secretion of CSF. In pathological states, excessive secretion of CSF by the CPE, or other dysfunctions mentioned above, leads to abnormal accumulation of CSF in the ventricles or subarachnoid space, and eventually to hydrocephalus. We describe the functional role of the CPE with the aim of suggesting its role in the formation of hydrocephalus and ultimately providing researchers with ideas to target the CPE as a therapeutic tool for hydrocephalus, in order to discover more effective means to alleviate or even cure hydrocephalus.

## Data Availability

Not applicable.

## Abbreviations

iNPH: Idiopathic normal pressure hydrocephalus
CSF: Cerebrospinal fluid
CPE: Choroid plexus epithelial
BIF: Brain interstitial fluid
CP: Choroid plexus
BCSFB: Blood–cerebrospinal fluid barrier
NKCC1: Na–K–2Cl co-transporter 1
NHE1: The Na/H exchanger 1
AE2: Anion exchanger2
AQP: Aquaporin
IVH: Intraventricular hemorrhage
PHVD: Post-hemorrhagic ventricular dilatation
SAH: Subarachnoid hemorrhage
ENaC: Epithelial Na + channel
KCC: K + –Cl− cotransporters
PMCA: Ca2 + -ATPase)
NCX: Na + /Ca2 + exchanger
TRPV4: Transient receptor potential vanilloid 4
PMAT: Plasma membrane monoamine transporter protein
AVP: Arginine pressor
NCBE: Na + -dependent Cl−/HCO3− exchanger
SPARC: Secreted protein acidic and rich in cysteine
PIT2: Pi transporter2
URAT1: Uric acid transporter protein
BCRP: Breast cancer resistance protein
RAGE: Receptor for advanced glycosylation end-products protein
IDE: Insulin-degrading enzymes
AVP: Arginine vasopressin
ANP: Atrial natriuretic peptide
ERKs: Extracellular signal-regulated kinases
TBI: Traumatic brain injury
